# Immunological effect of local ablation combined with immunotherapy on solid malignancies

**DOI:** 10.1186/s40880-017-0216-5

**Published:** 2017-06-07

**Authors:** Yusuke Takahashi, Noriyuki Matsutani, Takashi Nakayama, Hitoshi Dejima, Hirofumi Uehara, Masafumi Kawamura

**Affiliations:** Department of General Thoracic Surgery, Teikyo University School of Medicine, 2-11-1 Kaga, Itabashi, Tokyo, 173-8606 Japan

**Keywords:** Immunotherapy, Solid malignancies, Cryosurgery, Radiotherapy, Stereotactic body radiotherapy

## Abstract

Recent comprehensive investigations clarified that immune microenvironment surrounding tumor cells are deeply involved in tumor progression, metastasis, and response to treatment. Furthermore, several immunotherapeutic trials have achieved successful results, and the immunotherapeutic agents are available in clinical practice. To enhance their demonstrated efficacy, combination of immunotherapy and ablation has begun to emerge. Local ablations have considerable advantages as an alternative therapeutic option, especially its minimal invasiveness. In addition, local ablations have shown immune-regulatory effect in preclinical and clinical studies. Although the corresponding mechanisms are still unclear, the local ablations combined with immunotherapy have been suggested in the treatment of several solid malignancies. This article aims to review the published data on the immune-regulatory effects of local ablations including stereotactic body radiotherapy, cryoablation, radiofrequency ablation, and high-intensity-focused ultrasound. We also discuss the value of local ablations combined with immunotherapy. Local ablations have the potential to improve future patient outcomes; however, the effectiveness and safety of local ablations combined with immunotherapy should be further investigated.

## Background

Recently, comprehensive investigations have shown that immune components contribute to tumor progression and are strong predictors in patients with solid malignancies [1–7]. The recent success of immune checkpoint inhibitors such as programmed death-1 (PD-1) and programmed death-ligand 1 (PD-L1) blocking antibodies in clinical trials [8–13] greatly impacted treatment strategies of several malignancies. Subsequently, immunotherapies including chimeric antigen receptor (CAR) T cell therapy have been studied by ongoing clinical trials [14–18]. Immunotherapeutic approaches are recognized as a promising and expansible strategy; however, the clinical results of immunotherapies are not always satisfying [19, 20], and their effects need to be enhanced by facilitating a favorable immune microenvironment [21, 22].

Although local ablations including stereotactic body radiotherapy (SBRT) and cryoablation are relatively new, they have become alternative, minimally invasive local therapeutic options for several solid malignancies [23–28]. Advances in these novel ablations should be reevaluated in terms of possible immunological advantages [22, 29, 30]. In addition, recent studies have demonstrated the possible synergistic effect of these local ablations when combined with immunotherapy [31–33]. This raises the following questions: (1) whether combining local ablations with immunotherapy improves the survival outcomes of patients with solid tumors, (2) whether it is possible to differentiate the immunologic effect from abscopal effect of local ablations, and (3) which local ablation is the most likely to be effective when combined with immunotherapy. In answering these questions it would be ideal to focus on specific type(s) of cancer; however, there is a severe lack of data if our purview is limited to one specific type of solid tumor. To this end, we aim to review all published data available at PubMed on the immunological anti-tumor effects of local ablations and their possible additive effects when combined with immunotherapy.

## Local ablations

### Stereotactic body radiotherapy

Ablative radiation technique, termed SBRT, has been developed in the last few decades on the basis of knowledge and experience of stereotactic radio-surgery for brain tumors [34]. With image-guided target localization and multiple precision beams, SBRT minimizes damage to normal tissue around the tumor, which allows increased tumoricidal dose and reduced fractionation. Generally, local therapeutic effect of radiotherapy is well recognized as tumor cell death through DNA damage which renders proliferating tumor cells more sensitive to radiation than normal tissues [35]. Previous studies have mostly focused on DNA damage and the repair capacity of cells. In recent years, however, other molecular pathways contributing to cell stress, including those associated with immunologic anti-tumor effect, have been elucidated [36].

#### Immunological effect of irradiation

Several studies have investigated the changes in expression of major histocompatibility complex (MHC) class I and antigen presentation after irradiation both in vitro and in vivo [37–39]. Radiation beam exposure can enhance immune anti-tumor response through up-regulation of MHC class I in a dose-dependent manner [39]. The MHC class I up-regulation could be mediated by mammalian target of rapamycin (mTOR) activation and antigen presentation [39]. More recently, interferon beta was shown to stimulate MHC class I expression when exposed to radiation in combination with chemotherapeutic agents in vitro [40]. Gameiro et al. [41] suggested that radiation-induced release of high mobility group box 1 (HMGB1), one of the key regulators of systemic inflammatory response [42], activate antigen-presenting cells and subsequent antigen-specific T-cell response. Barrio et al. [43] demonstrated that the phagocytosis of dying, radiated tumor cells by both dendritic cells and macrophages could equally induced specific CD8^+^ T-cell cross presentation. In addition, the “abscopal effect” defined as tumor regression outside of irradiation field, was reported in patients treated with irradiation including SBRT [44]. Furthermore, a relatively higher dose of radiation per fraction may induce more significant anti-tumor immune effects in experimental models [45, 46]. Based on this data, the immunological anti-tumor effect of irradiation is potentially translatable to clinical practice and is the target of ongoing clinical trials [47, 48].

#### Effect of irradiation in combination with immunotherapy

Since several immune-regulatory agents including checkpoint inhibitors were shown to have significant efficacy on several solid malignancies [8–13], a combination strategy has now emerged. Previous literature has suggested the potential enhanced immunological anti-tumor effect of these agents in combination with irradiation [49, 50]. Local radiation-induced immune-regulatory effects may synergistically activate specific immunologic anti-tumor response and immune checkpoint inhibitors if they overcome immunosuppressive nature of local tumor microenvironment. In preclinical model, CD8^+^ T cells were considered to play a key role in regulating immune response by producing interferon gamma [51–53]. In another study, the immune-regulatory effect of radiotherapy in combination with an immune checkpoint inhibitor may be largely regulated through the mTOR pathway via MHC class I expression of tumor cells, dendritic cell activation, and CD8^+^ T cell function [54]. This suggested mechanism may activate an antigen-specific, systemic immune-regulatory response. Assuming the underlying mechanisms of the tumor microenvironment response to irradiation are similar, other local ablations may have the potential to replace irradiation when combined with immune-regulatory agents. Additionally, other classes of novel immune-regulatory agents might have synergistic effect with local radiotherapy. For instance, Toll-like receptor, which is expressed by antigen-presenting cells as well as effector B and T cells, is an alternative in combination with irradiation [54]. These classes of novel immune-regulatory agents in combination with SBRT may have the potential to drive even more efficient anti-tumor immunity.

### Cryoablation

Cryoablation therapy, which destroys tumor tissue through several cycles of extremely cold temperatures and thawing, has recently emerged as an option of minimally invasive treatment of various solid tumors [24]. It relies on controlled and local freezing, resulting in tissue damage by removing thermal energy from tumor cells. The subsequent necrosis and apoptosis are the basic mechanisms of cryoablation therapy [24]. The cryoablation “dose” is difficult to define differently from radiation dose, even though numerous preclinical and clinical reports attempted to elucidate “dose” needed for complete tumor destruction. On the other hand, thermal distribution was sharply distinguishable [55]. Despite this, the magnitude of tissue damage did not correlate to absolute temperatures [56]. There are multiple possible reasons for this discrepancy as many factors contribute to artificial freeze damage, such as the number of cycles, duration of freezing, nadir temperature, and the susceptibility of tumor cells.

The cell death is primarily induced by mechanical tissue injury through ice crystal formation. The cryo-probe, which is placed inside of tumor tissues, delivers extremely cold temperatures. Thus, the tissue damage depends on the distance from the cryo-probe to great vessels, which can largely affect degree of tissue temperature. Subsequent microcirculation failure leads to intracellular dehydration. Ice crystal formation also damages integrity of cell membrane [57]. Secondary intracellular ice crystal formation causes vascular stenosis and tissue ischemia that largely decrease cell viability. These two mechanisms synergistically contribute to tumor cell death. Cryoablation-induced cell deaths also relies on the apoptosis cascade which includes mitochondrial dysfunction mediated by decreased Bcl-2 expression and increased expression of Bax, a pro-apoptotic protein [58, 59].

Since the early 1970s, cryoablation has been thought to have abscopal immune-regulatory effects on remote tumors [60, 61]. However, another study reported that cryoablation alone did not always induce an abscopal effect and it may require specific circumstances to be present [62]. Recent literature showed that approximately three-fourths of tumor cell death by cryoablation may be caused by increased proinflammatory cytokine levels, which in turn contribute to decreased tumor growth rate and favorable survival outcomes, in mice [63]. Several clinical studies have further demonstrated similar findings that T-cell response was activated by increased serum cytokines [64, 65]. In addition, several researchers documented the increased immunological anti-tumor effect in combination with dendritic cells [66] as well as several immune-regulatory agents including lipopolysaccharide [67] and anti-cytotoxic T lymphocyte-associated antigen 4 (CTLA-4) antibody [68]. Taken together, these data suggest an immunological advantage and promising translatability of this ablative procedure. However, to date no clinical trials have investigated the efficacy of cryoablation in combination with immune-regulatory agents.

### Radiofrequency ablation

Radiofrequency ablation (RFA) has been applied to treat several solid malignancies such as kidney [69], breast [70], and liver cancers [71, 72]. It has also been applied to treat metastatic tumors of the liver [73–76]. This modality destroys target tumor tissue by applying thermal energy which leads to a temperature above 50 °C [77, 78]. The radiofrequency probe generates resistive heat by electric current, causing destruction of tissue [23]. Therefore, local therapeutic effects are largely affected by tumor size and RFA probe location. Clinically, RFA is mostly restricted to small-sized tumor especially those <3 cm in diameter [24].

While apoptotic cell death is not usually harmful for the host immune system, necrotic cell death releases signal molecules which can sometimes lead to dangerous reactions. Among the signal molecules, increased cell surface expression of heat shock proteins (HSPs) has been shown to activate dendritic cells [79], and several serum proinflammatory cytokines have been demonstrated to contribute to the clinical immunomodulation following RFA [80, 81].

Several preclinical studies have reported the potential effect of combining RFA with immunotherapy. CTLA-4 blocking antibody showed additive enhancement of antigen-specific CD8^+^ T-cell induction after RFA in vivo [82]. PD-1-blocking antibody was also reported to boost anti-tumor immunity elicited by RFA [83]. Antibody-conjugated interleukin-2 [84] as well as dendritic cell injection [85, 86] also enhanced the therapeutic effect of RFA in experimental models.

### High-intensity-focused ultrasound

High-intensity-focused ultrasound (HIFU) is a local ablation which applies acoustic cavitation without invasive procedures; it has been applied for solid tumors including breast cancer [87].

In previous literature, HIFU demonstrated antigen-specific immune-regulatory effect mediated by increased HSP-70 [87] and proinflammatory cytokines [88]. HIFU has garnered attention as a promising local ablation which may enhance anti-tumor immunity [33]. It may also have possible additive effect in combination with immunotherapy [33].

### Other ablative therapies

Microwave ablation provides high thermal energy by excitation of water molecules, causing tissue injury and tumor cell necrosis [89, 90], which has been clinically used to treat liver, lung, breast, and bone cancers [91]. Dendritic cell injection with microwave ablation improved anti-tumor immune cell subsets in patients with hepatocellular carcinoma [92].

Laser-induced thermal ablation causes coagulation necrosis by a refraction of laser light on the tumor tissue. This high-energy therapeutic modality led to tissue temperature over 60 °C [93]. In clinical observations, laser ablation increased the levels of serum proinflammatory cytokines including interleukin-6 in patients with liver tumors [94].

## Conclusions and perspectives

Recent advances in ablative engineering and its computed tomography-guided procedure have provided basis to conduct the minimally invasive local treatment of solid tumors. These ablations have been clinically used as alternative local approaches for treatment of various solid malignancies especially in unresectable or medically complexed cases. These modalities have secondary advantages including minimal invasiveness, patient comfort, and cosmetic outcome. This is in addition to the aforementioned immune-regulatory effects. It should be noted that the data reviewed in this article are mostly preclinical and there has been no definitive data to address whether local ablations in combination with immunotherapy can improve survival outcome of patients with solid tumors. The preclinical data reviewed in the current study may provide rationale for future clinical trial. SBRT in particular appears to demonstrate positive immunological advantages, and some clinical trials are evaluating its utility. However, further analysis is required to address which ablation is the most promising as no studies have compared the immunological advantages of different ablative modalities yet. Furthermore, the immune effect of local ablations may vary according to many factors related with tumor characteristics and treatment. Although the effectiveness and safety of combination of local ablations and immunotherapy should be investigated, they have the potential to improve further patient outcomes.

## Abbreviations

CAR: chimeric antigen receptor
SBRT: stereotactic body radiotherapy
MHC: major histocompatibility complex
HMGB1: high mobility group box 1
mTOR: mammalian target of rapamycin
TCLA-4: T lymphocyte-associated antigen 4
RFA: radiofrequency ablation
HSPs: heat shock proteins
HIFU: high-intensity-focused ultrasound

## References

[1] Chargin A, Morgan R, Sundram U, Shults K, Tsay EL, Ratti N (2016). Quantification of PD-L1 and PD-1 expression on tumor and immune cells in non-small cell lung cancer (NSCLC) using non-enzymatic tissue dissociation and flow cytometry. Cancer Immunol Immunother.

[2] Guislain A, Gadiot J, Kaiser A, Jordanova ES, Broeks A, Sanders J (2015). Sunitinib pretreatment improves tumor-infiltrating lymphocyte expansion by reduction in intratumoral content of myeloid-derived suppressor cells in human renal cell carcinoma. Cancer Immunol Immunother.

[3] Linardou H, Gogas H (2016). Toxicity management of immunotherapy for patients with metastatic melanoma. Ann Transl Med.

[4] Suzuki K, Kadota K, Sima CS, Nitadori J, Rusch VW, Travis WD (2013). Clinical impact of immune microenvironment in stage I lung adenocarcinoma: tumor interleukin-12 receptor β2 (IL-12Rβ2), IL-7R, and stromal FoxP3/CD3 ratio are independent predictors of recurrence. J Clin Oncol.

[5] Takahashi Y, Horio H, Hato T, Harada M, Matsutani N, Morita S (2015). Prognostic significance of preoperative neutrophil-lymphocyte ratios in patients with stage I non-small cell lung cancer after complete resection. Ann Surg Oncol.

[6] Takahashi Y, Kawamura M, Hato T, Harada M, Matsutani N, Horio H (2016). Neutrophil–lymphocyte ratio as a prognostic marker for lung adenocarcinoma after complete resection. World J Surg.

[7] Weide B, Allgaier N, Hector A, Forschner A, Leiter U, Eigentler TK (2015). Increased CCL17 serum levels are associated with improved survival in advanced melanoma. Cancer Immunol Immunother.

[8] Rittmeyer A, Barlesi F, Waterkamp D, Park K, Ciardiello F, von Pawel J, OAK Study Group (2017). Atezolizumab versus docetaxel in patients with previously treated non-small-cell lung cancer (OAK): a phase 3, open-label, multicentre randomised controlled trial. Lancet.

[9] Reck M, Rodríguez-Abreu D, Robinson AG, Hui R, Csőszi T, Fülöp A, KEYNOTE-024 Investigators (2016). Pembrolizumab versus chemotherapy for PD-L1-positive non-small-cell lung cancer. N Engl J Med.

[10] Ascierto PA (2015). Immunotherapies and novel combinations: the focus of advances in the treatment of melanoma. Cancer Immunol Immunother.

[11] Borghaei H, Paz-Ares L, Horn L, Spigel DR, Steins M, Ready NE (2015). Nivolumab versus docetaxel in advanced nonsquamous non-small-cell lung cancer. N Engl J Med.

[12] Motzer RJ, Escudier B, McDermott DF, George S, Hammers HJ, Srinivas S, CheckMate 025 Investigators (2015). Nivolumab versus everolimus in advanced renal-cell carcinoma. N Engl J Med.

[13] Weber JS, D’Angelo SP, Minor D, Hodi FS, Gutzmer R, Neyns B (2015). Nivolumab versus chemotherapy in patients with advanced melanoma who progressed after anti-CTLA-4 treatment (CheckMate 037): a randomised, controlled open-label, phase 3 trial. Lancet Oncol.

[14] Ahmed N, Brawley VS, Hegde M, Robertson C, Ghazi A, Gerken C (2015). Human epidermal growth factor receptor 2 (HER2)-specific chimeric antigen receptor-modified T Cells for the immunotherapy of HER2-positive sarcoma. J Clin Oncol.

[15] Holzinger A, Barden M, Abken H (2016). The growing world of CAR T cell trials: a systematic review. Cancer Immunol Immunother.

[16] Junghans RP, Ma Q, Rathore R, Gomes EM, Bais AJ, Lo AS (2016). Phase I trial of anti-PSMA designer CAR-T cells in prostate cancer: possible role for interacting interleukin 2-T cell pharmacodynamics as a determinant of clinical response. Prostate.

[17] Mayor M, Zeltsman M, McGee E, Adusumilli PS (2016). A regional approach for CAR T-cell therapy for mesothelioma: from mouse models to clinical trial. Immunotherapy.

[18] Suck G, Odendahl M, Nowakowska P, Seidl C, Wels WS, Klingemann HG (2016). NK-92: an ‘off-the-shelf therapeutic’ for adoptive natural killer cell-based cancer immunotherapy. Cancer Immunol Immunother.

[19] Hamanishi J, Mandai M, Ikeda T, Minami M, Kawaguchi A, Murayama T (2015). Safety and antitumor activity of anti-PD-1 antibody, nivolumab, in patients with platinum-resistant ovarian cancer. J Clin Oncol.

[20] Noguchi M, Moriya F, Koga N, Matsueda S, Sasada T, Yamada A (2016). A randomized phase II clinical trial of personalized peptide vaccination with metronomic low-dose cyclophosphamide in patients with metastatic castration-resistant prostate cancer. Cancer Immunol Immunother.

[21] Santegoets SJ, Stam AG, Lougheed SM, Gall H, Scholten PE, Reijm M (2013). T cell profiling reveals high CD4+ CTLA-4+ T cell frequency as dominant predictor for survival after prostate GVAX/ipilimumab treatment. Cancer Immunol Immunother.

[22] Twyman-Saint Victor C, Rech AJ, Maity A, Rengan R, Pauken KE, Stelekati E (2015). Radiation and dual checkpoint blockade activate non-redundant immune mechanisms in cancer. Nature.

[23] Azzam G, Lanciano R, Arrigo S, Lamond J, Ding W, Yang J (2015). SBRT: an opportunity to improve quality of life for oligometastatic prostate cancer. Front Oncol.

[24] Baust JG, Gage AA, Bjerklund Johansen TE, Baust JM (2014). Mechanisms of cryoablation: clinical consequences on malignant tumors. Cryobiology.

[25] Inoue M, Nakatsuka S, Jinzaki M (2014). Cryoablation of early-stage primary lung cancer. Biomed Res Int.

[26] Roach MC, Videtic GM, Bradley JD, IASLC Advanced Radiation Technology Committee (2015). Treatment of peripheral non-small cell lung carcinoma with stereotactic body radiation therapy. J Thorac Oncol.

[27] Salji M, Jones R, Paul J, Birrell F, Dixon-Hughes J, Hutchison C, Cryotherapy in Prostate Cancer (CROP) study team (2014). Feasibility study of a randomised controlled trial to compare (deferred) androgen deprivation therapy and cryotherapy in men with localised radiation-recurrent prostate cancer. Br J Cancer.

[28] Timmerman RD, Herman J, Cho LC (2014). Emergence of stereotactic body radiation therapy and its impact on current and future clinical practice. J Clin Oncol.

[29] Levy A, Chargari C, Marabelle A, Perfettini JL, Magné N, Deutsch E (2016). Can immunostimulatory agents enhance the abscopal effect of radiotherapy?. Eur J Cancer.

[30] Sharabi AB, Lim M, DeWeese TL, Drake CG (2015). Radiation and checkpoint blockade immunotherapy: radiosensitisation and potential mechanisms of synergy. Lancet Oncol.

[31] Confino H, Hochman I, Efrati M, Schmidt M, Umansky V, Kelson I (2015). Tumor ablation by intratumoral Ra-224-loaded wires induces anti-tumor immunity against experimental metastatic tumors. Cancer Immunol Immunother.

[32] Keisari Y, Hochman I, Confino H, Korenstein R, Kelson I (2014). Activation of local and systemic anti-tumor immune responses by ablation of solid tumors with intratumoral electrochemical or alpha radiation treatments. Cancer Immunol Immunother.

[33] van den Bijgaart RJ, Eikelenboom DC, Hoogenboom M, Fütterer JJ, den Brok MH, Adema GJ (2017). Thermal and mechanical high-intensity focused ultrasound: perspectives on tumor ablation, immune effects and combination strategies. Cancer Immunol Immunother.

[34] Vogl TJ, Eckert R, Naguib NN, Beeres M, Gruber-Rouh T, Nour-Eldin NA (2016). Thermal ablation of colorectal lung metastases: retrospective comparison among laser-induced thermotherapy, radiofrequency ablation, and microwave ablation. AJR Am J Roentgenol.

[35] Formenti SC, Demaria S (2009). Systemic effects of local radiotherapy. Lancet Oncol.

[36] Galluzzi L, Joza N, Tasdemir E, Maiuri MC, Hengartner M, Abrams JM (2008). No death without life: vital functions of apoptotic effectors. Cell Death Differ.

[37] Martín-Orozco E, Ferragut JA, Garcia-Peñarrubia P, Ferrer-Montiel A (2005). Acquisition of multidrug resistance by L1210 leukemia cells decreases their tumorigenicity and enhances their susceptibility to the host immune response. Cancer Immunol Immunother.

[38] Nikitina EY, Gabrilovich DI (2001). Combination of gamma-irradiation and dendritic cell administration induces a potent antitumor response in tumor-bearing mice: approach to treatment of advanced stage cancer. Int J Cancer.

[39] Reits EA, Hodge JW, Herberts CA, Groothuis TA, Chakraborty M, Wansley EK (2006). Radiation modulates the peptide repertoire, enhances MHC class I expression, and induces successful antitumor immunotherapy. J Exp Med.

[40] Wan S, Pestka S, Jubin RG, Lyu YL, Tsai YC, Liu LF (2012). Chemotherapeutics and radiation stimulate MHC class I expression through elevated interferon-beta signaling in breast cancer cells. PLoS ONE.

[41] Gameiro SR, Malamas AS, Bernstein MB, Tsang KY, Vassantachart A, Sahoo N (2016). Tumor cells surviving exposure to proton or photon radiation share a common immunogenic modulation signature, rendering them more sensitive to T cell-mediated killing. Int J Radiat Oncol Biol Phys.

[42] Takahashi Y, Matsutani N, Dejima H, Nakayama T, Okamura R, Uehara H (2016). Therapeutic potential of recombinant thrombomodulin for lung injury following pneumonectomy via inhibition of HMGB1 in mice. J Trauma Acute Care Surg.

[43] Barrio MM, Abes R, Colombo M, Pizzurro G, Boix C, Roberti MP (2012). Human macrophages and dendritic cells can equally present MART-1 antigen to CD8(+) T cells after phagocytosis of gamma-irradiated melanoma cells. PLoS ONE.

[44] Reynders K, Illidge T, Siva S, Chang JY, De Ruysscher D (2015). The abscopal effect of local radiotherapy: using immunotherapy to make a rare event clinically relevant. Cancer Treat Rev.

[45] Filatenkov A, Baker J, Mueller AM, Kenkel J, Ahn GO, Dutt S (2015). Ablative tumor radiation can change the tumor immune cell microenvironment to induce durable complete remissions. Clin Cancer Res.

[46] Park HJ, Griffin RJ, Hui S, Levitt SH, Song CW (2012). Radiation-induced vascular damage in tumors: implications of vascular damage in ablative hypofractionated radiotherapy (SBRT and SRS). Radiat Res.

[47] Reissfelder C, Timke C, Schmitz-Winnenthal H, Rahbari NN, Koch M, Klug F (2011). A randomized controlled trial to investigate the influence of low dose radiotherapy on immune stimulatory effects in liver metastases of colorectal cancer. BMC Cancer.

[48] Safi S, Beckhove P, Warth A, Benner A, Roeder F, Rieken S (2015). A randomized phase II study of radiation induced immune boost in operable non-small cell lung cancer (RadImmune trial). BMC Cancer.

[49] Postow MA, Callahan MK, Barker CA, Yamada Y, Yuan J, Kitano S (2012). Immunologic correlates of the abscopal effect in a patient with melanoma. N Engl J Med.

[50] Slovin SF, Higano CS, Hamid O, Tejwani S, Harzstark A, Alumkal JJ (2013). Ipilimumab alone or in combination with radiotherapy in metastatic castration-resistant prostate cancer: results from an open-label, multicenter phase I/II study. Ann Oncol.

[51] Derer A, Frey B, Fietkau R, Gaipl US (2016). Immune-modulating properties of ionizing radiation: rationale for the treatment of cancer by combination radiotherapy and immune checkpoint inhibitors. Cancer Immunol Immunother.

[52] Dovedi SJ, Adlard AL, Lipowska-Bhalla G, McKenna C, Jones S, Cheadle EJ (2014). Acquired resistance to fractionated radiotherapy can be overcome by concurrent PD-L1 blockade. Cancer Res.

[53] Kroon P, Gadiot J, Peeters M, Gasparini A, Deken MA, Yagita H (2016). Concomitant targeting of programmed death-1 (PD-1) and CD137 improves the efficacy of radiotherapy in a mouse model of human BRAFV600-mutant melanoma. Cancer Immunol Immunother.

[54] Verbrugge I, Gasparini A, Haynes NM, Hagekyriakou J, Galli M, Stewart TJ (2014). The curative outcome of radioimmunotherapy in a mouse breast cancer model relies on mTOR signaling. Radiat Res.

[55] Babaian RJ, Donnelly B, Bahn D, Baust JG, Dineen M, Ellis D (2008). Best practice statement on cryosurgery for the treatment of localized prostate cancer. J Urol.

[56] Robilotto AT, Baust JM, Van Buskirk RG, Gage AA, Baust JG (2013). Temperature-dependent activation of differential apoptotic pathways during cryoablation in a human prostate cancer model. Prostate Cancer Prostatic Dis.

[57] Gage AA, Baust JG (2007). Cryosurgery for tumors. J Am Coll Surg.

[58] Clarke DM, Baust JM, Van Buskirk RG, Baust JG (2004). Addition of anticancer agents enhances freezing-i*nduced prostate cancer cell d*eath: implications of mitochondrial involvement. Cryobiology.

[59] Forest V, Hadjeres R, Bertrand R, Jean-François R (2009). Optimisation and molecular signalling of apoptosis in sequential cryotherapy and chemotherapy combination in human A549 lung cancer xenografts in SCID mice. Br J Cancer.

[60] Alblin RJ, Soanes WA, Gonder MJ (1971). Prospects for cryo-immunotherapy in cases of metastasizing carcinoma of the prostate. Cryobiology.

[61] Gursel E, Roberts M, Veenema RJ (1972). Regression of prostatic cancer following sequential cryotherapy to the prostate. J Urol.

[62] Urano M, Tanaka C, Sugiyama Y, Miya K, Saji S (2003). Antitumor effects of residual tumor after cryoablation: the combined effect of residual tumor and a protein-bound polysaccharide on multiple liver metastases in a murine model. Cryobiology.

[63] Takahashi Y, Izumi Y, Matsutani N, Dejima H, Nakayama T, Okamura R (2016). Optimized magnitude of cryosurgery facilitating anti-tumor immunoreaction in a mouse model of Lewis lung cancer. Cancer Immunol Immunother.

[64] Si T, Guo Z, Hao X (2008). Immunologic response to primary cryoablation of high-risk prostate cancer. Cryobiology.

[65] Thakur A, Littrup P, Paul EN, Adam B, Heilbrun LK, Lum LG (2011). Induction of specific cellular and humoral responses against renal cell carcinoma after combination therapy with cryoablation and granulocyte-macrophage colony stimulating factor: a pilot study. J Immunother.

[66] Alteber Z, Azulay M, Cafri G, Vadai E, Tzehoval E, Eisenbach L (2014). Cryoimmunotherapy with local co-administration of ex vivo generated dendritic cells and CpG-ODN immune adjuvant, elicits a specific antitumor immunity. Cancer Immunol Immunother.

[67] Ismail M, Morgan R, Harrington K, Davies J, Pandha H (2010). Immunoregulatory effects of freeze injured whole tumour cells on human dendritic cells using an in vitro cryotherapy model. Cryobiology.

[68] Waitz R, Solomon SB, Petre EN, Trumble AE, Fassò M, Norton L (2012). Potent induction of tumor immunity by combining tumor cryoablation with anti-CTLA-4 therapy. Cancer Res.

[69] Mimura H, Arai Y, Yamakado K, Sone M, Takeuchi Y, Miki T (2016). Phase I/II study of radiofrequency ablation for malignant renal tumors: Japan Interventional Radiology in Oncology Study Group 0701. Cardiovasc Intervent Radiol.

[70] Peek MC, Ahmed M, Napoli A, Usiskin S, Baker R, Douek M (2016). Minimally invasive ablative techniques in the treatment of breast cancer: a systematic review and meta-analysis. Int J Hyperthermia.

[71] Thomasset SC, Dennison AR, Garcea G (2015). Ablation for recurrent hepatocellular carcinoma: a systematic review of clinical efficacy and prognostic factors. World J Surg.

[72] Yu SJ, Yoon JH, Lee JM, Lee JY, Kim SH, Cho YY (2016). Percutaneous ethanol injection therapy is comparable to radiofrequency ablation in hepatocellular carcinoma smaller than 1.5  cm: a matched case-control comparative analysis. Medicine.

[73] Lee BC, Lee HG, Park IJ, Kim SY, Kim KH, Lee JH (2016). The role of radiofrequency ablation for treatment of metachronous isolated hepatic metastasis from colorectal cancer. Medicine.

[74] Liu B, Zhou L, Huang G, Zhong Z, Jiang C, Shan Q (2015). First experience of ultrasound-guided percutaneous ablation for recurrent hepatoblastoma after liver resection in children. Sci Rep.

[75] Du S, Ni J, Weng L, Ma F, Li S, Wang W (2015). Aggressive locoregional treatment improves the outcome of liver metastases from grade 3 gastroenteropancreatic neuroendocrine tumors. Medicine.

[76] Seidensticker M, Garlipp B, Scholz S, Mohnike K, Popp F, Steffen I (2015). Locally ablative treatment of breast cancer liver metastases: identification of factors influencing survival (the Mammary Cancer Microtherapy and Interventional Approaches (MAMMA MIA) study). BMC Cancer.

[77] Mertyna P, Hines-Peralta A, Halpern E, Goldberg W, Goldberg SN (2007). Radiofrequency ablation: variability in heat sensitivity in tumors and tissues. J Vasc Interv Radiol.

[78] Wood M, Goldberg S, Lau M, Goel A, Alexander D, Han F (2011). Direct measurement of the lethal isotherm for radiofrequency ablation of myocardial tissue. Circ Arrhythm Electrophysiol.

[79] Schueller G, Kettenbach J, Sedivy R, Stift A, Friedl J, Gnant M (2004). Heat shock protein expression induced by percutaneous radiofrequency ablation of hepatocellular carcinoma in vivo. Int J Oncol.

[80] Fietta AM, Morosini M, Passadore I, Cascina A, Draghi P, Dore R (2009). Systemic inflammatory response and down modulation of peripheral CD25+ Foxp3+ T-regulatory cells in patients undergoing radiofrequency thermal ablation for lung cancer. Hum Immunol.

[81] Jansen MC, van Wanrooy S, van Hillegersberg R, Rijken AM, van Coevorden F, Prevoo W (2008). Assessment of systemic inflammatory response (SIR) in patients undergoing radiofrequency ablation or partial liver resection for liver tumors. Eur J Surg Oncol.

[82] den Brok MH, Sutmuller RP, van der Voort R, Bennink EJ, Figdor CG, Ruers TJ (2004). In situ tumor ablation creates an antigen source for the generation of antitumor immunity. Cancer Res.

[83] Shi L, Chen L, Wu C, Zhu Y, Xu B, Zheng X (2016). PD-1 blockade boosts radiofrequency ablation-elicited adaptive immune responses against tumor. Clin Cancer Res.

[84] Johnson EE, Yamane BH, Buhtoiarov IN, Lum HD, Rakhmilevich AL, Mahvi DM (2009). Radiofrequency ablation combined with KS-IL2 immunocytokine (EMD 273066) results in an enhanced antitumor effect against murine colon adenocarcinoma. Clin Cancer Res.

[85] Dromi SA, Walsh MP, Herby S, Traughber B, Xie J, Sharma KV (2009). Radiofrequency ablation induces antigen-presenting cell infiltration and amplification of weak tumor-induced immunity. Radiology.

[86] Nakagawa H, Mizukoshi E, Iida N, Terashima T, Kitahara M, Marukawa Y (2014). In vivo immunological antitumor effect of OK-432-stimulated dendritic cell transfer after radiofrequency ablation. Cancer Immunol Immunother.

[87] Wu F, Wang ZB, Cao YD, Zhou Q, Zhang Y, Xu ZL (2007). Expression of tumor antigens and heat-shock protein 70 in breast cancer cells after high-intensity focused ultrasound ablation. Ann Surg Oncol.

[88] Deng J, Zhang Y, Feng J, Wu F (2010). Dendritic cells loaded with ultrasound-ablated tumour induce in vivo specific antitumour immune responses. Ultrasound Med Biol.

[89] Sun Y, Wang Y, Ni X, Gao Y, Shao Q, Liu L (2009). Comparison of ablation zone between 915- and 2450-MHz cooled-shaft microwave antenna: results in in vivo porcine livers. AJR Am J Roentgenol.

[90] Xu Y, Shen Q, Wang N, Wu PP, Huang B, Kuang M (2017). Microwave ablation is as effective as radiofrequency ablation for very-early-stage hepatocellular carcinoma. Chin J Cancer.

[91] Sharma R, Wagner JL, Hwang RF (2011). Ablative therapies of the breast. Surg Oncol Clin N Am.

[92] Yu MA, Liang P, Yu XL, Han ZY, Dong XJ, Wang YU (2015). Multiple courses of immunotherapy with different immune cell types for patients with hepatocellular carcinoma after microwave ablation. Exp Ther Med.

[93] Robinson DS, Parel JM, Denham DB, González-Cirre X, Manns F, Milne PJ (1998). Interstitial laser hyperthermia model development for minimally invasive therapy of breast carcinoma. J Am Coll Surg.

[94] Kallio R, Sequeiros R, Surcel HM, Ohtonen P, Kiviniemi H, Syrjälä H (2006). Early cytokine responses after percutaneous magnetic resonance imaging guided laser thermoablation of malignant liver tumors. Cytokine.

